# HIV related stigma associated with social support, alcohol use disorders, depression, anxiety, and suicidal ideation among people living with HIV: a systematic review and meta-analysis

**DOI:** 10.1186/s13033-022-00527-w

**Published:** 2022-03-04

**Authors:** Bahram Armoon, Marie-Josée Fleury, Amir-Hossein Bayat, Yadollah Fakhri, Peter Higgs, Ladan Fattah Moghaddam, Leila Gonabadi-Nezhad

**Affiliations:** 1 Douglas Mental Health University Institute, Research Centre, 6875 LaSalle Boulevard, Montreal, QC H4H 1R3 Canada; 2 Department of Psychiatry, McGill University, 33 Pine Avenue West, Montreal, QC H3A 1A1 Canada; 3 Social Determinants of Health Research Center, Saveh University of Medical Sciences, Saveh, Iran; 4 Food Health Research Center, Hormozgan University of Medical Sciences, Bandar Abbas, Iran; 5 Department of Public Health, La Trobe University, Melbourne, Australia; 6 Burnet Institute, Melbourne, Victoria Australia; 7 Department of Nursing, Faculty of Nursing and Midwifery, Tehran Medical Sciences, Islamic Azad University, Tehran, Iran; 8 Department of Psychiatry, Faculty of Medicine, Aja University of Medical Sciences, Tehran, Iran

**Keywords:** Stigma, Anxiety, Suicidal ideation, Depression

## Abstract

**Background:**

Stigma is a social phenomenon known to have a negative impact on the lives of people living with HIV (PLWH). However, defining HIV-related stigma (HRS) is difficult because of the intersection it has with structural inequalities, and cultural differences, discrimination by health care providers that measure stigma among PLWH. HIV/AIDS has been characterized as a traumatic experience and PLWH may experience stigma which can cause negative mental health disorders and experiences, including emotional distress, shame, depression, anxiety, suicidal ideation. A systematic review of the evidence on the mental disorders of PLWH is currently lacking. This study aimed to analyze the association between HRS and social support, alcohol use disorders and mental health disorders and experiences (depression, anxiety, and suicidal ideation) among PLWH.

**Methods:**

In accordance with Preferred Reporting Items for Systematic Reviews and Meta-Analyses (PRISMA) this study searched PubMed, Scopus, Web of sciences, PsycInfo, SciELO and Cochrane library electronic databases to identify publications between January 1992 and August 2020 that discussed social support, alcohol use disorders, mental health disorders and experiences (i.e., depression and anxiety and suicidal ideation) associated with HRS. Pooled Odds Ratios (ORs) were utilized at a 95% confidence level, and as sampling methods differed between articles pooled estimates used a random effects model.

**Results:**

Twenty-two studies with 9548 participants met the eligibility criteria. No association was observed between HRS and alcohol use disorders. PLWH who had higher levels of social supports were less likely to report HRS. Participants who had been diagnosed with anxiety were 1.89 times more likely to report HRS, while those diagnosed with depression were 1.61 times more. Respondents who reported suicidal ideation also were 1.83 times more likely to report HRS.

**Conclusions:**

This meta-analysis supports that HRS has a detrimental association with anxiety, depression and suicidal ideation, but social support protects again HRS in PLWH. Applying interventions which focus on the mental health disorders of PLWH may decrease HRS. Provision of social support by practitioners, combined with mental health treatment and assessments, and designing methods to identify stigma at different stages of illness are warranted.

## Background

Stigma and discrimination are among the major issues confronted by people living with HIV (PLWH) [1, 2]. HIV-related stigma (HRS) has been specified as discrediting and discriminating against PLWH [3]. PLWH often face stigma [4, 5]. Types of stigma that PLWH may experience include perceived, enacted, anticipated and internalized stigma [4, 6]. Perceived stigma is defined by the discrimination experienced by PLWH, and may include prejudicial attitudes from people in the society [7]. Enacted stigma includes any externally stigmatizing reaction that may lead to the unfairly or negative treatment to stigmatized individuals [8], anticipated stigma, is the negative treatments that people believe the others may do if their stigmatized condition is proved [9] and internalized stigma is the agreement with negative stereotypes and self-negative societal attitudes of the persons [10]. Discrimination against people with HIV can be considered one form of human rights violation. Other violations include the rights to health, the right to work, the right to have a family, the right to privacy, the right to insurance and social security [11]. PLWH may also be faced with limits or denial of access to health care [12].

Stigma may originate from misunderstandings about HIV transmission and from judgmental attitudes towards those social groups that are affected by HIV [13–15]. Studies have reported HIV discrimination in healthcare settings including denial of care or treatment, HIV testing without adequate consent, confidentiality violations, negative beliefs and other humiliating practices by healthcare workers [16, 17].

Previous studies reported that PLWH who had HRS experiences indicated lower levels of perceived social support [18, 19]. Social support is defined as the procurement of psychological and material resources by people in their social network [20]. According to the reports, social resources are also accessible for PLWH who are more vulnerable to stigma, this has public health consequences since social support is associated with powerful health advantages for these people [21]. The benefits include less depression [22], positive health behaviors such as adherence to treatments [23], improved quality of life [24], and slower disease progression [25].

HIV/AIDS has been characterized as a traumatic experience and PLWH may experience stigma which can cause mental health disorders and experiences, including emotional distress [26–28], shame [29], depression [30], anxiety [31, 32] and suicidal ideation [33, 34]. Depression, anxiety, and suicidal ideation may have negative influences on HIV treatment and adherence, and the prognosis of HIV infection [35, 36]. PLWH are 7–36 times more likely to experience suicidal ideation and attempt compared to the general public [37, 38]. Studies concerning health outcomes associated with HIV stigma, reported that alcohol use is increased among patients with HIV who experienced greater stigma [39, 40]. Alcohol use disorders among people with HIV-related stigma is a major concern knowing that high alcohol use is associated with numerous harmful consequences such as risky sexual behaviors that may increase the likelihood of HIV transmission [41–43]. Also, PLWH who have alcohol use disorders are approximately 50–60% less likely to be adherent to antiretroviral therapy (ART) [42]. Moreover, studies confirmed that alcohol use disorders are associated with lower CD4 cell count [44], higher cigarette smoking [45], substance use disorders [45], depression disorder [46], anxiety disorder [47] and suicidal ideation [48] among PLWH.

To our knowledge no study has conducted systematic reviews and meta-analysis concerning the association between HRS and social support, alcohol use disorders and mental health disorders and experiences (depression, anxiety, and suicidal ideation). Two previous meta-analysis studies on HRS have been published, Logie and Gadalla investigated the demographic factors associated with HRS [1] and Rueda et al. [3] considered HRS together with specific health outcomes such as quality of life and physical health. The present study is distinct from the previous studies in several aspects. The first difference is that rather than considering all aspects of physical and mental health this study specifically examines social support, alcohol use disorders and mental health disorders and experiences including, depression, anxiety, and suicidal ideation. The second difference is that the findings of this study are more generalizable as it used an odds ratio (OR) approach controlling for confounding as compared to previous research which was conducted using a correlational approach which cannot illustrate causal relationships. Given this, a better understanding of the outcomes of stigma on social support, alcohol use disorders and mental health disorders and experiences may improve HIV treatment management strategies including enhanced coping strategies, compliance, and adherence to treatment for PLWH. The aims of the present study were to investigate the association between HRS and social support, alcohol use disorders and mental health disorders and experiences (depression, anxiety, and suicidal ideation) among PLWH. The study hypothesizes that: HRS is inversely associated with social support whereas, HRS is positively associated with alcohol use disorders and mental health disorders and experiences among PLWH.

## Methods

### Search strategy and study selection

We conducted a systematic search of databases (PubMed, Scopus, Web of sciences, PsycInfo, SciELO and Cochrane library) for peer-reviewed papers published between January 1992 and August 2020. Each database was searched for mesh and non‐mesh terms concerning HIV/AIDS and stigma (Table 1). Studies were selected from across four WHO regions—including 12 studies from Region of the Americas (n = 5581 participants), one from the European Region (n = 381 participants), three from the African Region (n = 1380 participants) and six from the Western Pacific Region (n = 9877 participants). The USA had the highest number of included studies (nine studies, 3717 participants). Considering the World Bank country income levels, our sample includes 11 studies (n = 5350) from high-income countries, 9 studies (n = 11,224) from an upper middle income country, and 2 studies (n = 645) from low income economic countries.

**Table 1:** Search strategy

PubMed search		
Search number	Query	Item founds
#17	((((((((Social Support[MeSH Terms]) OR (Alcohol Drinking[MeSH Terms])) OR (Depression[MeSH Terms])) OR (Anxiety[MeSH Terms])) OR (Suicidal Ideation[MeSH Terms]))) AND (HIV[MeSH Terms])) AND ((((((((Social Stigma[MeSH Terms]) OR (stigma[Title/Abstract])) OR (shame[MeSH Terms])) OR (Self Disclosure[MeSH Terms])) OR (Self Concept[MeSH Terms])) OR (Negative Self-Image[Title/Abstract])) OR (blame[Title/Abstract])) OR (feel guilty[Title/Abstract]))) AND (people who lived with HIV[Title/Abstract]) AND (people living with HIV [Title/Abstract])	
#16	(((((((Social Stigma[MeSH Terms]) OR (stigma[Title/Abstract])) OR (shame[MeSH Terms])) OR (Self Disclosure[MeSH Terms])) OR (Self Concept[MeSH Terms])) OR (Negative Self-Image[Title/Abstract])) OR (blame[Title/Abstract])) OR (feel guilty[Title/Abstract])	
#15	feel guilty[Title/Abstract]	
#14	blame[Title/Abstract]	
#13	Negative Self-Image[Title/Abstract]	
#12	Self Concept[MeSH Terms]	
#11	Self Disclosure[MeSH Terms]	
#10	shame[MeSH Terms]	
#9	people who lived with HIV[Title/Abstract]	
#8	stigma[Title/Abstract]	
#7	Social Stigma[MeSH Terms]	
#6	HIV[MeSH Terms]	
#5	Suicidal Ideation[MeSH Terms]	
#4	Anxiety[MeSH Terms]	
#3	Depression[MeSH Terms]	
#2	Alcohol Drinking[MeSH Terms]	
#1	Social Support[MeSH Terms]	

Scopus search		
	TITLE (social AND support)	
#1	TITLE (alcohol AND drinking)	
#2	TITLE (depression)	
#3		
#4	TITLE (hiv)	
#5	TITLE (social AND stigma)	
#6	TITLE (stigma)	
#7	TITLE (people AND who AND lived AND with AND hiv)	
#8	TITLE (people AND living AND with AND hiv)	
#9	TITLE (self AND disclosure)	
#10	TITLE (self AND concept)	
#11	TITLE (negative AND self-image)	
#12	TITLE (feel AND guilty)	
#13	(TITLE (social AND stigma)) OR (TITLE (stigma))	
#14	(TITLE (people AND who AND lived AND with AND hiv)) OR (TITLE (living AND with AND hiv))	
#15	(TITLE (social AND support)) OR (TITLE (alcohol AND drinking)) OR (TITLE (depression)) OR (TITLE (anxiety)) OR (TITLE (suicidal AND ideation)) OR (TITLE (self AND disclosure)) OR (TITLE (self AND concept)) OR (TITLE (negative AND self-image)) OR (TITLE (blame)) OR (TITLE (feel AND guilty))	
#16	((TITLE (social AND support)) OR (TITLE (alcohol AND drinking)) OR (TITLE (depression)) OR (TITLE (anxiety)) OR (TITLE (suicidal AND ideation)) OR (TITLE (self AND disclosure)) OR (TITLE (self AND concept)) OR (TITLE (negative AND self-image)) OR (TITLE (blame)) OR (TITLE (feel AND guilty))) AND (TITLE (hiv)) AND ((TITLE (people AND who AND lived AND with AND hiv)) OR (TITLE (people AND living AND with AND hiv)))	

Cochrane search		
ID	Search	
#1	MeSH descriptor: [Social Support] explode all trees	
#2	MeSH descriptor: [Alcohol Drinking] explode all trees	
#3	MeSH descriptor: [Depression] explode all trees	
#4	MeSH descriptor: [Anxiety] explode all trees	
#5	MeSH descriptor: [Suicidal Ideation] explode all trees	
#6	MeSH descriptor: [HIV] explode all trees	
#7	MeSH descriptor: [Social Stigma] explode all trees	
#8	(stigma):ti (Word variations have been searched)	
#9	(people who lived with HIV):ti (Word variations have been searched)	
#10	(Living with HIV):ti (Word variations have been searched)	
#11	MeSH descriptor: [Shame] explode all trees	
#12	MeSH descriptor: [Self Disclosure] explode all trees	
#13	MeSH descriptor: [Self Concept] explode all trees	
#14	(Negative Self-Image):ti (Word variations have been searched)	
#15	(blame):ti (Word variations have been searched)	
#16	(feel guilty):ti (Word variations have been searched)	
#17	#9 OR #10	
#18	#1 OR #2 OR #3 OR #4 OR #5	
#19	#7 OR #8 OR #11 OR #12 OR #13 OR #14 OR #15 OR #16	
#20	#18 AND #6 AND #17 AND #19	
#21	#18 AND #17 AND #19	

Web of knowledge		
#19	#18 AND #17 AND #16 Indexes = SCI-EXPANDED, SSCI, A&HCI, CPCI-S, CPCI-SSH, BKCI-S, BKCI-SSH, ESCI, CCR-EXPANDED, IC Timespan = All years	
#18	#9 OR #8 Indexes = SCI-EXPANDED, SSCI, A&HCI, CPCI-S, CPCI-SSH, BKCI-S, BKCI-SSH, ESCI, CCR-EXPANDED, IC Timespan = All years	
#17	#15 OR #14 OR #13 OR #12 OR #11 OR #10 Indexes = SCI-EXPANDED, SSCI, A&HCI, CPCI-S, CPCI-SSH, BKCI-S, BKCI-SSH, ESCI, CCR-EXPANDED, IC Timespan = All years	
#16	#5 OR #4 OR #3 OR #2 OR #1 Indexes = SCI-EXPANDED, SSCI, A&HCI, CPCI-S, CPCI-SSH, BKCI-S, BKCI-SSH, ESCI, CCR-EXPANDED, IC Timespan = All years	
#15	TI = (feel guilty) Indexes = SCI-EXPANDED, SSCI, A&HCI, CPCI-S, CPCI-SSH, BKCI-S, BKCI-SSH, ESCI, CCR-EXPANDED, IC Timespan = All years	
#14	TI = (blame) Indexes = SCI-EXPANDED, SSCI, A&HCI, CPCI-S, CPCI-SSH, BKCI-S, BKCI-SSH, ESCI, CCR-EXPANDED, IC Timespan = All years	
#13	TI = (Negative Self-Image) Indexes = SCI-EXPANDED, SSCI, A&HCI, CPCI-S, CPCI-SSH, BKCI-S, BKCI-SSH, ESCI, CCR-EXPANDED, IC Timespan = All years	
#12	TI = (Self Concept) Indexes = SCI-EXPANDED, SSCI, A&HCI, CPCI-S, CPCI-SSH, BKCI-S, BKCI-SSH, ESCI, CCR-EXPANDED, IC Timespan = All years	
#11	TI = (Self Disclosure) Indexes = SCI-EXPANDED, SSCI, A&HCI, CPCI-S, CPCI-SSH, BKCI-S, BKCI-SSH, ESCI, CCR-EXPANDED, IC Timespan = All years	
#10	TI = (Shame) Indexes = SCI-EXPANDED, SSCI, A&HCI, CPCI-S, CPCI-SSH, BKCI-S, BKCI-SSH, ESCI, CCR-EXPANDED, IC Timespan = All years	
#9	TI = (Living with HIV) Indexes = SCI-EXPANDED, SSCI, A&HCI, CPCI-S, CPCI-SSH, BKCI-S, BKCI-SSH, ESCI, CCR-EXPANDED, IC Timespan = All years	
#8	TI = (people who lived with HIV) Indexes = SCI-EXPANDED, SSCI, A&HCI, CPCI-S, CPCI-SSH, BKCI-S, BKCI-SSH, ESCI, CCR-EXPANDED, IC Timespan = All years	
#7	TI = (Stigma) Indexes = SCI-EXPANDED, SSCI, A&HCI, CPCI-S, CPCI-SSH, BKCI-S, BKCI-SSH, ESCI, CCR-EXPANDED, IC Timespan = All years	
#6	TI = (Social Stigma) Indexes = SCI-EXPANDED, SSCI, A&HCI, CPCI-S, CPCI-SSH, BKCI-S, BKCI-SSH, ESCI, CCR-EXPANDED, IC Timespan = All years	
#5	TI = (Suicidal Ideation) Indexes = SCI-EXPANDED, SSCI, A&HCI, CPCI-S, CPCI-SSH, BKCI-S, BKCI-SSH, ESCI, CCR-EXPANDED, IC Timespan = All years	
#4	TI = (Anxiety) Indexes = SCI-EXPANDED, SSCI, A&HCI, CPCI-S, CPCI-SSH, BKCI-S, BKCI-SSH, ESCI, CCR-EXPANDED, IC Timespan = All years	
#3	TI = (Depression) Indexes = SCI-EXPANDED, SSCI, A&HCI, CPCI-S, CPCI-SSH, BKCI-S, BKCI-SSH, ESCI, CCR-EXPANDED, IC Timespan = All years	
#2	TI = (Alcohol Drinking) Indexes = SCI-EXPANDED, SSCI, A&HCI, CPCI-S, CPCI-SSH, BKCI-S, BKCI-SSH, ESCI, CCR-EXPANDED, IC Timespan = All years	
#1	TI = (Social Support) Indexes = SCI-EXPANDED, SSCI, A&HCI, CPCI-S, CPCI-SSH, BKCI-S, BKCI-SSH, ESCI, CCR-EXPANDED, IC Timespan = All years	

PsycINFO search		
	(“Social Support” OR “Alcohol Drinking OR Depression OR” Anxiety” OR” Suicidal Ideation”) AND (“Shame” OR “Self-Disclosure” OR “Self-Concept” OR “Negative Self-Image” OR “blame” OR “feel guilty”) AND (“people who lived with HIV” OR “Living with HIV”)	

Scielo search		
	Social Support [Title words] or Alcohol Drinking [Title words] or Depression [Title words] or Anxiety [Title words] or Suicidal Ideation [Title words]and Shame [Title words] or Self-Disclosure [Title words] or Self-Concept [Title words] or Negative Self-Image [Title words] or blame [Title words] or feel guilty [Title words] and people who lived with HIV [Title words] or Living with HIV [Title words]	

### Inclusion/exclusion criteria

In this meta-analysis, we included observational studies (i.e., cross-sectional and cohort studies exploring the association between HRS and social support, alcohol use disorders and mental health disorders and experiences (depression, anxiety, and suicidal ideation) among PLWH. Characteristics that we considered for inclusion and exclusion criteria are shown in the Table 2.

**Table 2:** Inclusion and exclusion criteria

Inclusion criteria	Exclusion criteria
Study had to be published in English (between January 1992 and August 2020)	Study did not consider any systematic reviews and meta-analysis
Study had to have the sample constitute of people living with HIV as the main condition	Study did not consider any qualitative research
Study had to document HIV-related stigma as a factor associated with social support, alcohol use disorders, and common and serious mental disorders e.g., depression, anxiety, suicidal ideation	
Study had to have multivariable analysis as analytic methods	

### Screening and data extraction

To facilitate the process of selection, we used a Newcastle–Ottawa Scale (NOS) [49] recommended by the Cochrane Collaboration [50] addressing the selection criteria (Table 3). First, we reviewed the titles and abstracts of the selected studies. Second, to determine the studies’ general applicability for review, we retrieved and evaluated the full-texts of the collected papers. Two independent researchers conducted all the above-mentioned stages. Reviewers resolved any disagreements by consensus. Implementing κ-statistic, we measured inter-rater reliability at 0.65, highlighting substantial agreement between the reviewers. Data extraction included: publication year, the study location, the author’s name, the design of the study, the statistical analysis method, the study sample size, the key statistical data and any outcome measures.

**Table 3:** Risk of bias assessment using Newcastle–Ottawa scale

Study	Selection (***)	Comparability (*)	Exposure/outcome (*)	Method of assessment	Quality assessment	Quality assessment score
Levi-Minzi and Surratt [71]	***	*	*	Newcastle–Ottawa scale adapted for cross-sectional studies	Very good	5
Emlet et al. [67]	**	*	*	Newcastle–Ottawa scale adapted for cross-sectional studies	Good	4
Turan et al. [77]	***	*	*	Newcastle–Ottawa scale adapted for cross-sectional studies	Very good	5
Li et al. [72]	***	*	*	Newcastle–Ottawa scale adapted for cross-sectional studies	Very good	5
Zhang et al. [82]	*	*	*	Newcastle–Ottawa scale adapted for cross-sectional studies	Satisfactory	3
Zhang et al. [80]	***		*	Newcastle–Ottawa scale adapted for cross-sectional studies	Good	4
Courtenay–Quirk et al. [66]	**	*	*	Newcastle–Ottawa scale adapted for cross-sectional studies	Good	4
Zeng et al. [79]	*	*	*	Newcastle–Ottawa scale adapted for cross-sectional studies	Satisfactory	3
Li et al. [106]	***	*	*	Newcastle–Ottawa scale adapted for cross-sectional studies	Very good	5
Lambert et al. [65]	**	*	*	Newcastle–Ottawa scale adapted for cross-sectional studies	Good	4
Felker-Kantor et al. [69]	**	*	*	Newcastle–Ottawa scale adapted for cross-sectional studies	Good	4
Ferlatte et al. [70]	**		*	Newcastle–Ottawa scale adapted for cross-sectional studies	Satisfactory	3
Fekete et al. [68]	***	*	*	Newcastle–Ottawa scale adapted for cross-sectional studies	Very good	5
Galvan et al. [18]	**	*	*	Newcastle–Ottawa scale adapted for cross-sectional studies	Good	4
Akena et al. [63]	**		*	Newcastle–Ottawa scale adapted for cross-sectional studies	Satisfactory	3
Lee et al. [30]	***		*	Newcastle–Ottawa scale adapted for cross-sectional studies	Good	4
Pearson et al. [74]	**	*	*	Newcastle–Ottawa scale adapted for cross-sectional studies	Good	4
Burke et al. [64]	*	*	*	Newcastle–Ottawa scale adapted for cross-sectional studies	Satisfactory	3
Rael and Hampanda [76]	**	*	*	Newcastle–Ottawa scale adapted for cross-sectional studies	Good	4
Turan et al. [78]	**	*	*	Newcastle–Ottawa scale adapted for cross-sectional studies	Good	4
Peltzer and Ramlagan [75]	***	*	*	Newcastle–Ottawa scale adapted for cross-sectional studies	Very good	5
Zhang et al. [81]	***	*	*	Newcastle–Ottawa scale adapted for cross-sectional studies	Very good	5

### Quality appraisal

The quality appraisal tool was derived from the NOS [51–53]. We rated each study in terms of exposure, outcome, and comparability with a scale of very good, good, satisfactory and unsatisfactory quality domains as suggested by the Newcastle–Ottawa Scale. This scale consists of three domains (selection, comparability and exposure/outcome) and each domain included 3, 1 and 1 item respectively. Selection domain: (a) representativeness of the exposed group, (b) selection of the non-exposed group, and (c) ascertainment of exposure. Comparability domain is referring to comparability of groups on the basis of the design or analysis and exposure/outcome domain is referring to assessment of outcome. If a publication had each item, it got a score or star. A maximum of five for the quality scores was obtained by adding the items. Publications with a total score of 0–2, 3, 4 and 5 points were recorded as “unsatisfactory,” “satisfactory,” “good” or “very good” respectively.” Eventually, a very good, good and satisfactory quality comparability domain score identified the studies after controlling for ≥ 1 potential sociodemographic confounder characteristic (e.g., age, gender, income level), and unadjusted potential confounds (e.g., bivariate analysis). Two independent researchers assessed the quality of the included articles.

### Instruments

Berger Stigma Scale [54], HIV Stigma Measure [55], Internalized AIDS-Related Stigma Scale [56], and Demi-HIV Stigma Scale were the most frequently applied tools for measuring HIV-related stigma. The scales, including the Center for Epidemiologic Studies Depression Scale and the Brief Symptom Inventory (BSI) depression subscale [57], the BSI anxiety subscale [57], state anxiety [58], and the Symptom Check List-90-R [59], were employed to measure depression and anxiety. The Social Support Questionnaire [60] and the Medical Outcomes Study—Social Support Subscale [61] were employed to measure social support. Alcohol use disorders was assessed by the Alcohol Use Disorders Test (AUDIT) questionnaire [62] and participants were asked if they had ever thought to suicide.

### Statistical methods summary

The meta-analysis was carried out by generating pooled odds ratios (OR) and 95% confidence intervals (CIs) on identifying social support, alcohol use disorders and mental health disorders and experiences including, depression, anxiety, suicidal ideation related to HRS among PLWH. The OR was calculated applying a 2 × 2 table, and OR < 1 indicated a positive association between independent variables and HRS. An OR > 1 indicates a protective association between variables. An inverse variance weighting was implemented to compute summary effect sizes; these values were indicated by regression coefficients for the multivariate analyses.

Moreover, there was a variation in true effect sizes among the studies with random effects models; thus, a random-effects model was employed to conduct model selection and publication bias meta-analyses. Accordingly, we considered two uncertainty sources; within-study sampling error and between-study variance. The large Cochran’s Q statistics with small p-values and large I^2^ statistics were employed to suggest the heterogeneity in true effect sizes across the articles. The meta-analysis for studies that consisted of ≥ 10 articles were assessed in terms of publication bias for a specific outcome variable. Publication bias was evaluated by Funnel plots, trim-and-fill analysis, and Rosenthal’s fail-safe number.

## Results:

### Study characteristics

**Fig. 1:**
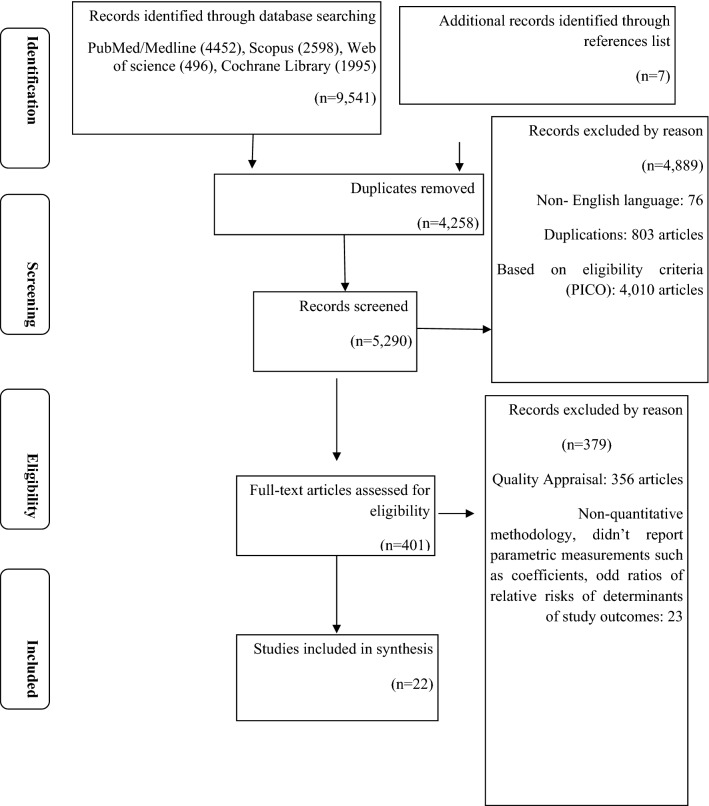
PRISMA flow diagram

The study selection process is shown in Fig. 1. 9,548 papers were found through 6 databases with additional manual searches of the reference lists of selected articles which is presented in Fig. 1 illustrating each stage of the study selection process. Of 9548 potentially relevant studies retained for screening, 401 were selected for full-text assessment, and 22 met the eligibility criteria for the review [18, 30, 63–82].

The meta-analysis included seven studies [67, 71, 72, 77, 80–82] that examined the association between social support and HIV related stigma among PLWH. Three studies were completed in high-income countries [67, 71, 77], and four studies were from upper middle-income countries [72, 80–82]. Studies were completed between 2014 and 2018, and the sample sizes ranged from 203 to 2987. All of four studies used a cross –sectional design. Three studies [69, 81, 82] evaluated the association of alcohol use disorders on HIV related stigma among PLWH. These studies had alcohol use disorders as an exposure variable and HIV related stigma among PLWH as an outcome variable. The studies were completed between 2016 and 2019 with sample sizes between 380 and 2987. Two studies were conducted in upper middle income countries [81, 82] and one in the USA (high income country) [69]. All the considered studies used cross-sectional analysis and evaluated the association of alcohol use disorders as a current measure using participant self-report.

Seventeen studies [18, 30, 63–65, 67–69, 71, 74–76, 78–82] examined the association of depression on HIV related stigma among PLWH. Eight studies were conducted in high-income countries [18, 30, 65, 67–69, 71, 78], seven studies in upper middle-income countries [64, 75, 76, 79–82], one study from a lower middle income country [74] and one study was conducted in a low income country [63]. Data were collected between 2002 and 2019 and the sample sizes of the studies ranged from 181 to 2987 participants and all studies were cross-sectional. Six studies [30, 69, 73, 80–82] examined the association between anxiety and HIV related stigma among PLWH. Two studies [30, 69] were conducted in high-income countries and four in an upper middle income countries [73, 80–82]. The dates of studies ranged from 2002 to 2019, and the sample sizes were between 239 and 2987. All six studies used a cross-sectional design. Two studies [66, 70] examined the association between suicidal ideation and HIV related stigma among PLWH. Both of them were conducted in high-income countries [66, 70]. The dates of studies ranged from 2006 to 2017, and the sample sizes were between 456 and 673. Both studies used a cross-sectional design to recruit participants.

### Results of the meta-analysis

We assessed the association between HRS and social support, alcohol use disorders and mental health disorders and experiences (depression, anxiety, and suicidal ideation) among PLWH (see Table 4). Plots 2–6 represent the results found.

**Table 4:** Main characteristics of the studies selected

Author	Participants	Year of publish	Sample size	Year of implementation	Country	Design	Quality of the evidence
Levi-Minzi and Surratt [71]	PLWHA	2014	503	2014	USA	Cross-section	Very Good
Emlet et al. [67]	PLWHA	2014	960	2013	Canada	Cross-section	Good
Turan et al. [77]	PLWHA	2018	203	2018	USA	Cross-section	Very Good
Li et al. [72]	MSM	2016	266	2014	China	Cross-section	Very Good
Zhang et al. [82]	PLWHA	2018	2987	2012–2015	China	Cross-section	Satisfactory
Zhang et al. [80]	PLWHA	2016	2987	2012–2013	China	Cross-section	Good
Courtenay–Quirk et al. [66]	MSM	2006	456	2006	USA	Cross-section	Good
Zeng et al. [79]	PLWHA	2018	411	2013	China	Cross-section	Satisfactory
Li et al. [106]	PLWHA	2018	239	2014	China	Cross-section	Very Good
Lambert et al. [65]	PLWHA	2019	355	2018	USA	Cross-section	Good
Felker-Kantor et al. [69]	PLWHA	2019	380	2015–2017	USA	Cross-section	Good
Ferlatte et al. [70]	MSM	2017	673	2014–2015	Canada	Cross-section	Satisfactory
Fekete et al. [68]	PLWHA	2018	181	2017	USA	Cross-section	Very Good
Galvan et al. [18]	PLWHA	2010	283	2005–2010	USA	Cross-section	Good
Akena et al. [63]	PLWHA	2012	368	2012	Uganda	Cross-section	Satisfactory
Lee et al. [30]	PLWHA	2002	268	2002	USA	Cross-section	Good
Pearson et al. [74]	PLWHA	2009	277	2009	Mozambicans	Cross-section	Good
Burke et al. [64]	PLWHA	2015	381	2012–2013	Russia	Cross-section	Satisfactory
Rael and Hampanda [76]	PLWHA	2016	231	2014	Mexico	Cross-section	Good
Turan et al. [78]	PLWHA	2017	1356	2014–2016	USA	Cross-section	Good
Peltzer and Ramlagan [75]	PLWHA	2011	735	2007–2008	South African	Cross-section	Very Good
Zhang et al. [81]	PLWHA	2016	2987	2012–2013	China	Cross-section	Very Good

### The association of HRS and alcohol use disorders among PLWH

**Fig. 2:**
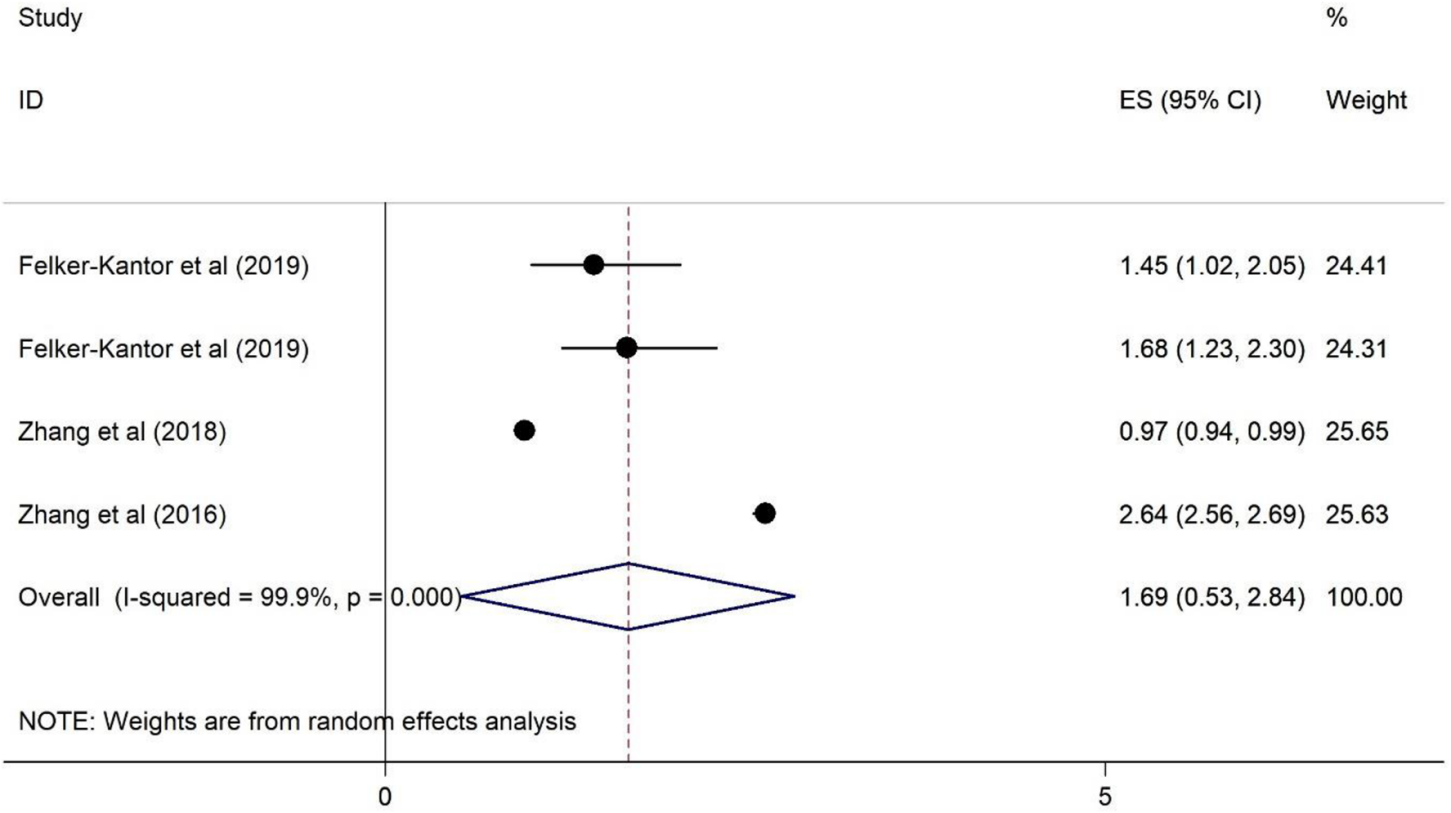
Forest plots for the association of HRS and alcohol use disorder among PWLH

As illustrated in Fig. 2, alcohol use disorders do not have any association on HRS among PLWH (OR = 1.69, 95% CI 0.53–2.84). The overall heterogeneity was 99.9%.

### The association of HRS and social support among PLWH

**Fig. 3:**
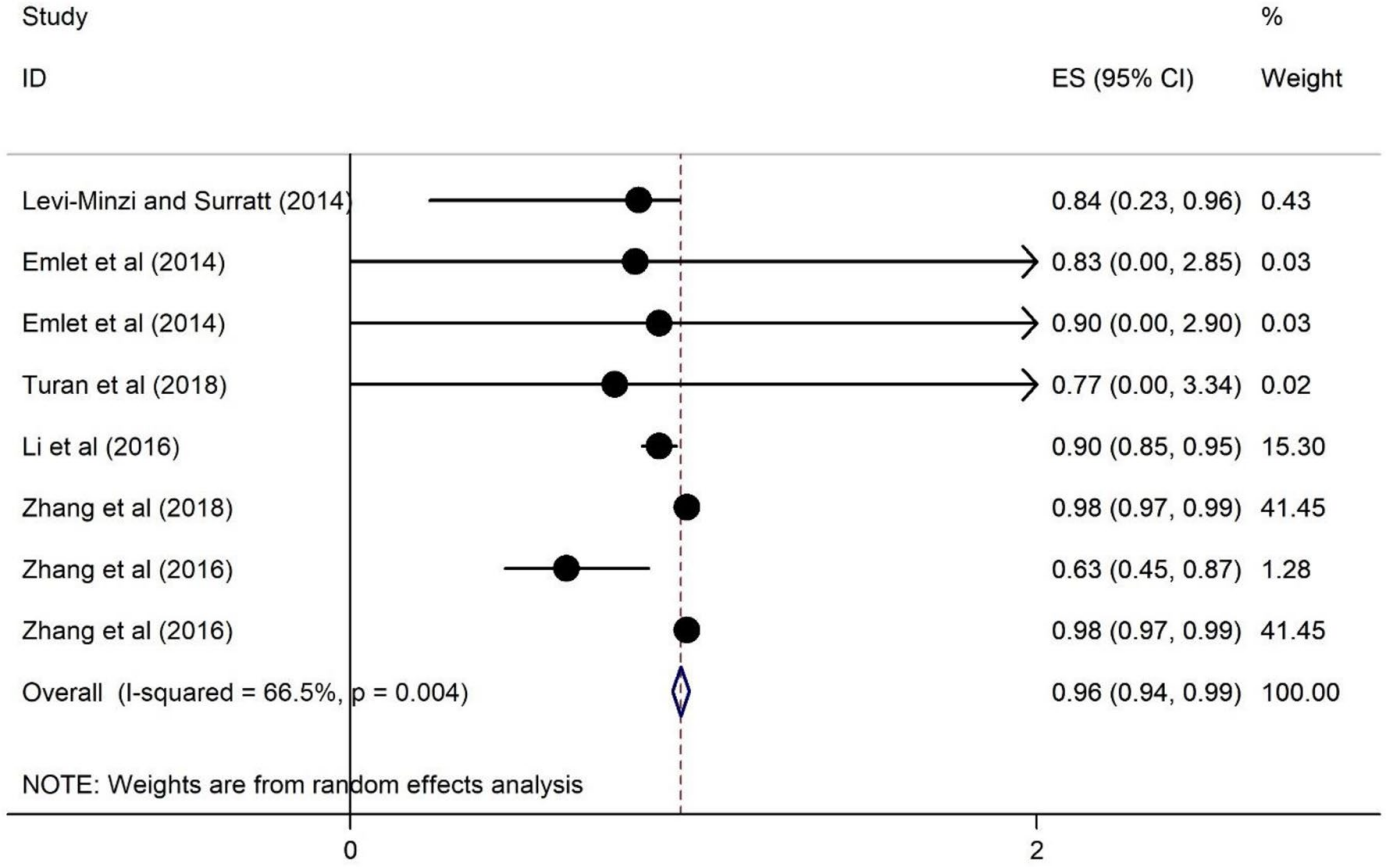
Forest plots for the association of HRS and higher social support among PWLH

The association of HRS and social support among PLWH are presented in Fig. 3. Those who had greater HRS were 0.96 times less likely to report higher social support (OR = 0.96, 95% CI 0.94, 0.99) and the heterogeneity is about 66.9% (Fig. 3).

### The association of HRS and anxiety among PLWH

**Fig. 4:**
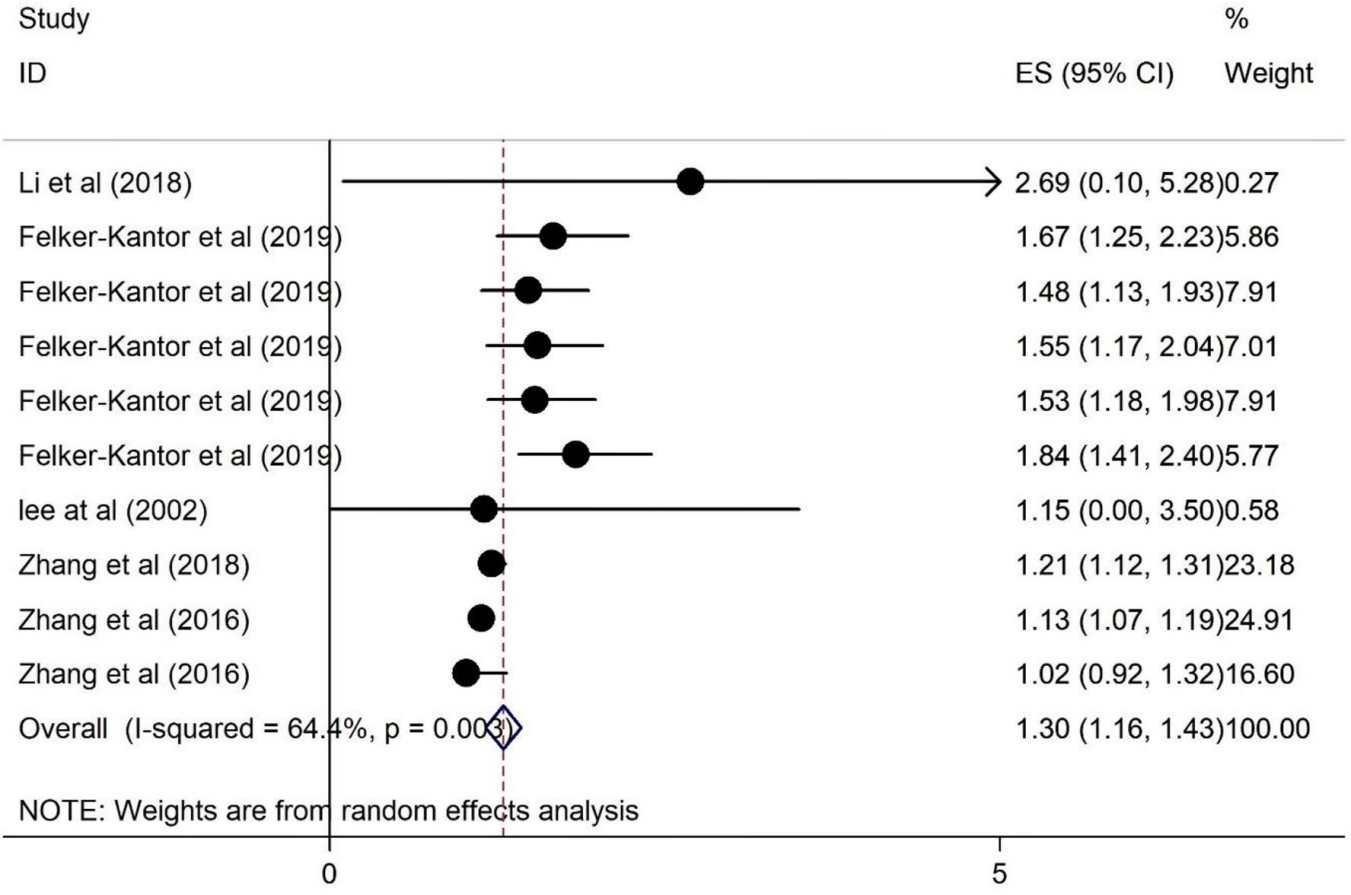
Forest plots for the association of HRS and anxiety among PWLH

Figure 4 presents the forest plot outlining the association of HRS and anxiety among PLWH. As illustrated in Fig. 4, HRS was positively associated with anxiety among the participants. The heterogeneity statistic is 64.4%, and the pooled effect size implies an association. PLWH reporting HRS were 1.89 times more likely to also report anxiety (OR, 1.30; 95% CI 1.16–1.43).

### The association of HRS and depression among PLWH

**Fig. 5:**
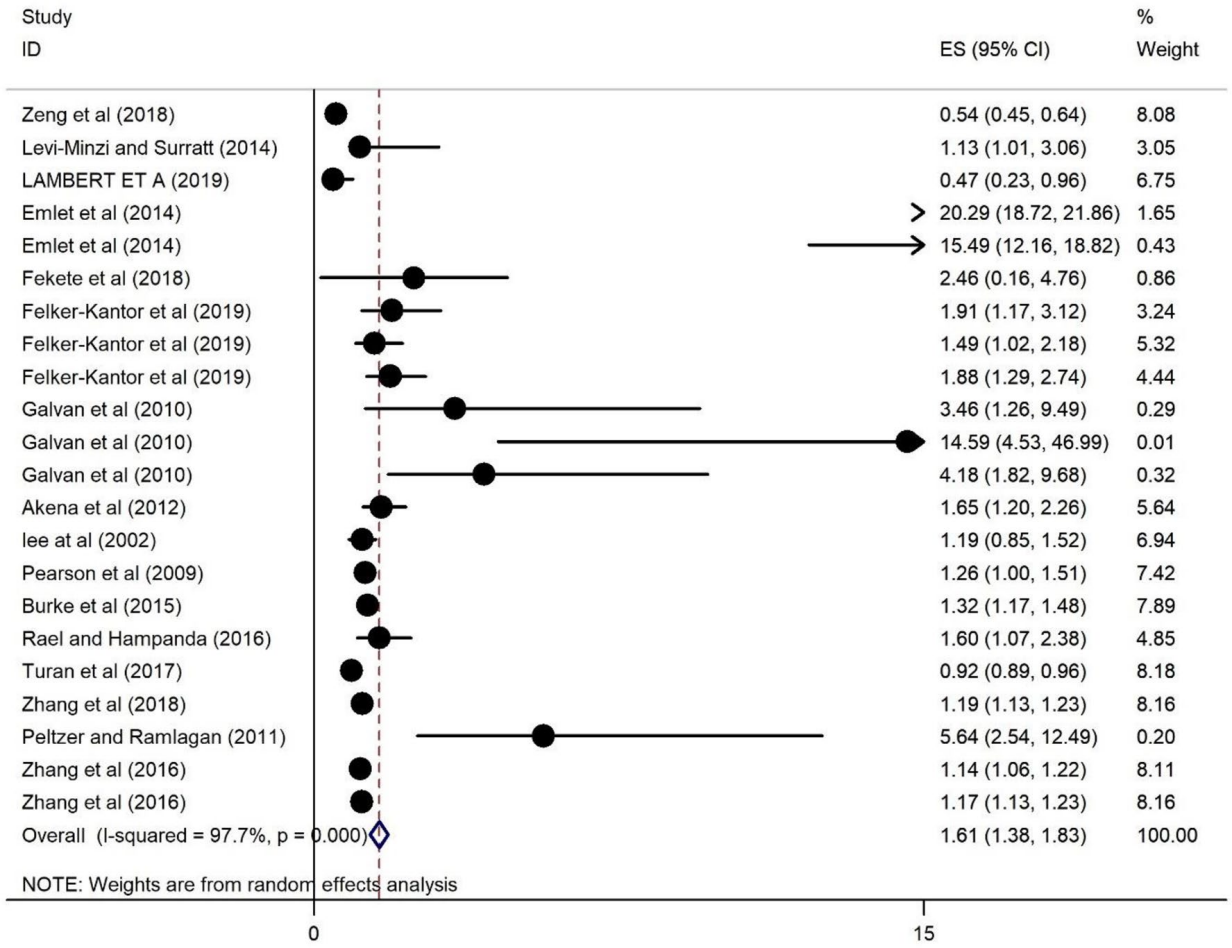
Forest plots for the association of HRS and depression among PWLH

The association of HRS and depression among PLWH are presented in Fig. 5 and show a positive association. Those PLWH living with HRS were 1.61 times more likely to report depression (OR = 1.61, 95% CI 1.38, 1.83) and the heterogeneity is 97.7%.

### The association of HRS and suicidal ideation among PLWH

**Fig. 6:**
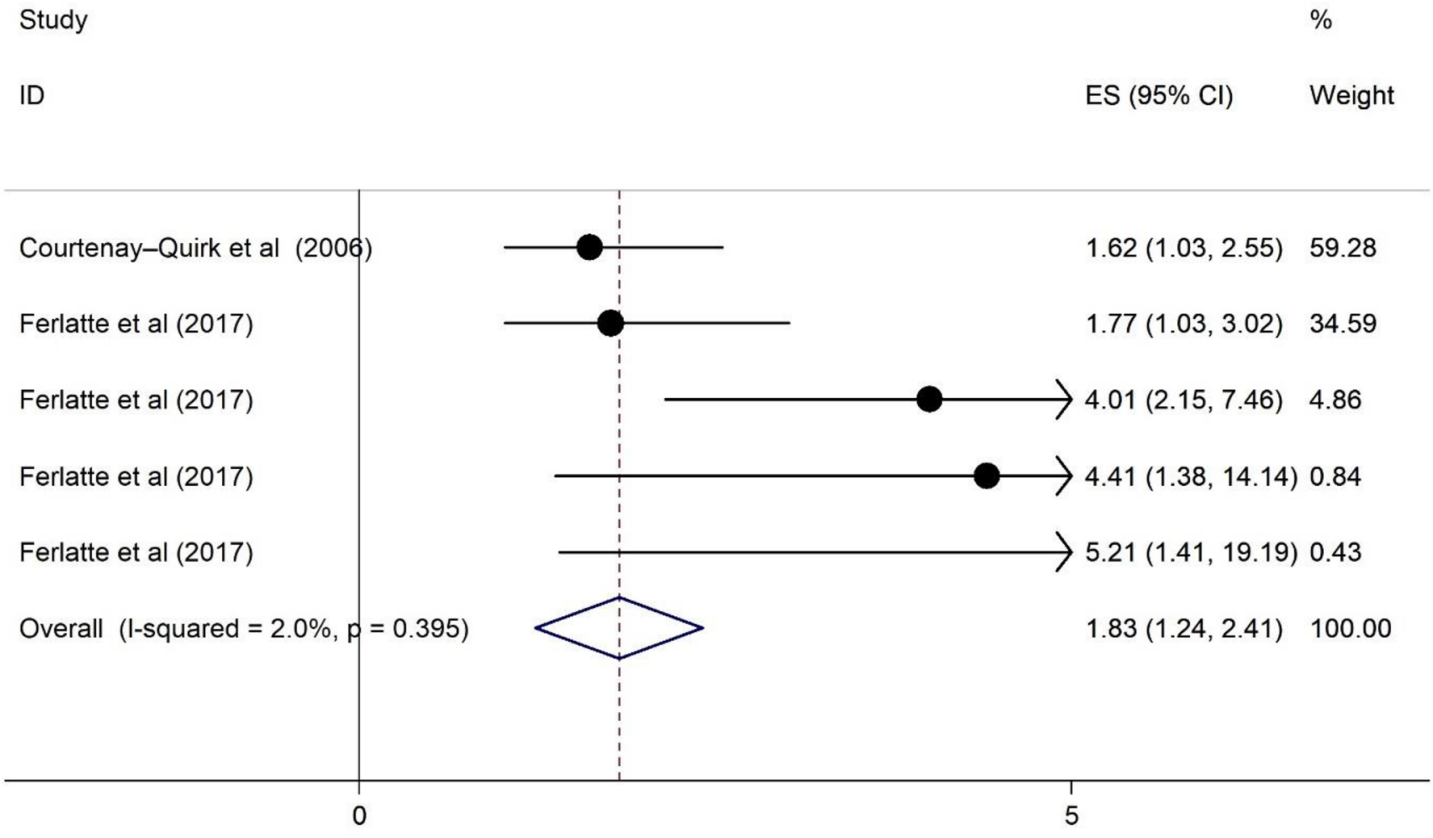
Forest plots for the association of HRS and suicidal ideation among PWLH

As illustrated in Fig. 6, HRS has a positive association on suicidal ideation among PLWH. The heterogeneity statistic is about 2.0%, and the pooled effect size implies a relative neutral association. Those respondents who reported HRS were 1.83 times more likely to have had suicidal ideation (OR = 1.83, 95% CI 1.24, 2.41). See Fig. 6.

### Publication bias

According to publication bias test, a significant publication bias among studies was noted. The publication bias was significant in social support (C = − 3.61, P value < 0.001), alcohol use disorders (C = − 3.32, P value = 0.052) and in mental health disorders and experiences subgroup including depression (C = 34.60, P value = 0.02), anxiety (C = 2.56, P value = 0.027) and suicidal ideation (C = 4.18, P value = 0.15) (See Table 5). Begg’s test and Egger’s test showed evidence of publication bias (Begg’s test: P < 0.001; Egger’s test: P < 0.001). Therefore, metatrim analysis was performed in order to remove the effect of publication bias on the pooled OR (OR = 0.17, 95% CI 0.13–0.21).

**Table 5:** Findings of publication bias using Eggers test

Suicidal ideation	C = 4.18, P value = 0.15
Depression	C = 34.60, P value = 0.02
Anxiety	C = 2.56, P value = 0.027
Alcohol	C = -3.32, P value = 0.052
Social support	C = -3.61, P value < 0.001

## Discussion

This meta-analysis reports on the association between HRS and social support, alcohol use disorders and mental health disorders and experiences (depression, anxiety, and suicidal ideation) among PLWH. A positive association was found between HRS and increased levels of depression, anxiety, suicidal ideation, and a negative association between social support and stigma.

Social support has a significant role in the psychological adjustment of people who have HIV/AIDS. PLWH who receive poor social support may encounter more difficulty adjusting to psychological issues by themselves. Previous studies showed that those PLWH with poor social support were at high risk for suicidal behaviors as measured by self-report [83–85]. Social support may reduce the negative feelings related to HIV stigma among PLWH, and also improves the belonging feelings [21, 86]. Social support accessibility provides relevant information and guidance of HIV treatment to PLWH [87, 88]. HIV stigma may reduce social networks and social interactions because of self-imposed social isolation and avoiding of negative judgment and guilt related to HIV [87]. Studies revealed that the sharing of positive HIV status with family, friends, or healthcare staff may lead to social stigma [54] and isolation or exclusion by community [1, 89]. Data also suggested that the lack of social support may induce depression in PLWH. Therefore, social support has a significant role in the psychological adjustment of PLWH. Improving family counseling and support services such as care for PLWH and promoting HIV screening among high-risk populations should be explored by both policy makers and health care workers.

The results of the present study showed that there was no association between alcohol use disorders and HRS. One possible reason for this is there is limited research examining the association between alcohol use disorders and HRS and more heterogeneous research in this area is needed. This study’s hypotheses were based on previous studies which indicated that higher levels of HRS were directly associated with alcohol use disorders [90, 91]. The reason may be due to that risky alcohol use among PLWH may increase risks for HIV transmission through reduced inhibitions which may lead to more risky sexual behavior and increased HIV transmission and progression [90]. Also, HRS may lead to important outcomes such as feelings of shame, fear of disclosure, isolation, and despair on HIV risks and reduce ART adherence in PLWH [91]. The obtained association between alcohol dependence and HIV stigma may explain that alcohol use disorders, as a HIV infection consequence [92], may increase the stigma. Also, alcohol use disorders might precede HIV infection and be increased by HIV stigma. Therefore, high HIV stigma may decrease the coping ability with the disease; or alternatively, alcohol use may increase the vulnerability of HIV-related stigma and discrimination [93]. It is recommended to increase health education about alcohol use disorder and its negative effect in the health institutions and the community to decrease the disorder. In addition, it is essential to screen for alcohol use disorders among patients with HIV/AIDS.

Furthermore, the results of present study showed that HRS is significantly associated with higher levels of both anxiety and depression. The findings of this present study are consistent with previous studies [94, 95]. Studies demonstrated that increased HRS was significantly associated with greater odds of higher levels of both anxiety and depression symptoms [94, 95]. Previous studies recommended a variety of coping strategies to manage the impact of HRS on depression. In this regard, the stigma-depression association was known to be moderated using disengagement coping style (avoidant strategies, i.e., disengagement from the stressor, denial, or wishful thinking) [96, 97]. Receiving enough social resources may impede adverse psychological responses to stressful situations; therefore, high levels of social support may decrease the negative effects of HRS, such as anxiety and depression, among PLWH [98].

Finally, HRS was significantly related with suicidal ideation and it is consistent with the results of other studies [38, 99]. The negative association of stigma may increase the psychological pressure experienced by PLWH and may increase suicidal ideation. Findings from a Nepalese study with PLWH indicated that depressive symptoms, rather than suicidal ideation were positively associated with higher levels of internalized stigma [100]. They also found that both perceived stigma and spiritual beliefs were the significant factors for suicidal ideation and this could be managed by preventive approaches to decreasing suicidal ideation. Additionally, suicide risk may be diminished through the perceived availability of social support, which could have a positive influence on health and mood among PLWH [101]. Coverage of mental health conditions in health insurance policies and reduce access to lethal means among persons at risk of suicide may lead to decreasing suicidal ideation among PLWH and also enhance their mental health and overall wellness. This gap of knowledge should be addressed by conducting longitudinal studies on the effects of other psychosocial factors. The negative effects of stigma may worsen the psychological pressure for PLWH, and in turn this may then impact suicidal ideation, because of the physical and emotional pressure felt from the discrimination.

### Strengths and limitations of the study

The study limitations included first relying on HRS self-reports. Second, most of the included studies were cross-sectional meaning causal and temporal association for all results were not possible. (However, this meta-analysis may enhance the statistical inference of analyses and increase the reliability of the evidence thus possibly mitigating this limitation). Also, since we did not interfere with the setting of independent and dependent variables, we had to report only the data that were published in the articles. One of the key strengths of the present meta-analysis was that it examined many different variables such as social support, alcohol use disorders and mental health disorders and experiences (depression, anxiety, and suicidal ideation) associated with HRS among PLWH. Additionally, every observational study was evaluated, regardless of geographical location or date of publication. Moreover, this is the first meta-analysis that specifically examines social support, alcohol use disorders and mental health disorders and experiences. Finally, the findings of this study are more generalizable as an OR approach controlling for confounding and illustrate causal relationships has been used.

### Implications for practice

In the present study, the findings showed that higher severity of psychosocial health problems was associated with greater HRS among PLWH. The most important advantage of the present study is the integration of different psychosocial health problems to develop a comprehensive study for the preliminary evaluation of HRS. The data from this meta-analysis could be implemented to inform health care workers about the psychosocial health problems of HRS.

## Conclusion

This meta-analysis supports that HRS has a detrimental association with anxiety, depression and suicidal ideation, but social support protects against HRS in PLWH. Applying interventions which focus on the mental health disorders of PLWH may decrease HRS. Provision of social support by practitioners [102], combined with mental health treatment and assessments, and designing methods to identify stigma at different stages of illness are warranted. Interventions aimed directly at health care practitioners may improve competence, non-judgment, confidentiality and awareness of the correlates of HRS [103, 104]. Interventions to enable PLWH to challenge existing stigma through skills and leadership training, taking part in conferences and advisory boards may also go some way decreasing HIV related stigma and ultimately to improving mental health.

HRS may lead to social and economic marginalization, therefore any attempts to decrease stigma and discrimination related to HIV/AIDS may assist countries to reach key targets for universal access. This will also be important to help protect and improve protection of human rights, as well as enhance respect for PLWH and other affected groups, and finally decrease the transmission of HIV [105].

## Data Availability

The datasets used and/or analyzed during the current study are available from the corresponding author on reasonable request.

## Abbreviations

PLWH: People living with HIV
HRS: HIV-related stigma
ART: Antiretroviral therapy
CI: Confidence intervals
NOS: Newcastle–Ottawa Scale
OR: Odds ratio
WHO: World Health Organization
BSI: Brief symptom inventory
AUDIT: Alcohol use disorders test

## References

[1] Logie C, Gadalla TM (2009). Meta-analysis of health and demographic correlates of stigma towards people living with HIV. AIDS Care.

[2] Armoon B, Higgs P, Fleury M-J, Bayat A-H, Moghaddam LF, Bayani A, Fakhri Y (2021). Socio-demographic, clinical and service use determinants associated with HIV related stigma among people living with HIV/AIDS: a systematic review and meta-analysis. BMC Health Serv Res.

[3] Rueda S, Mitra S, Chen S, Gogolishvili D, Globerman J, Chambers L, Wilson M, Logie CH, Shi Q, Morassaei S (2016). Examining the associations between HIV-related stigma and health outcomes in people living with HIV/AIDS: a series of meta-analyses. BMJ Open.

[4] Thomas BE, Rehman F, Suryanarayanan D, Josephine K, Dilip M, Dorairaj VS, Swaminathan S (2005). How stigmatizing is stigma in the life of people living with HIV: a study on HIV positive individuals from Chennai, South India. AIDS Care.

[5] Heckman TG, Heckman BD, Kochman A, Sikkema KJ, Suhr J, Goodkin K (2002). Psychological symptoms among persons 50 years of age and older living with HIV disease. Aging Ment Health.

[6] Bayat AH, Mohammadi R, Moradi-Joo M, Bayani A, Ahounbar E, Higgs P, Hemmat M, Haghgoo A, Armoon B (2020). HIV and drug related stigma and risk-taking behaviors among people who inject drugs: a systematic review and meta-analysis. J Addict Dis.

[7] Zeng C, Li L, Hong YA, Zhang H, Babbitt AW, Liu C, Li L, Qiao J, Guo Y, Cai W (2018). A structural equation model of perceived and internalized stigma, depression, and suicidal status among people living with HIV/AIDS. BMC Public Health.

[8] Gray AJ (2002). Stigma in psychiatry. J R Soc Med.

[9] Quinn DM, Earnshaw VA (2013). Concealable stigmatized identities and psychological well-being. Soc Pers Psychol Compass.

[10] Boyle MP (2018). Enacted stigma and felt stigma experienced by adults who stutter. J Commun Disord.

[11] Mukoyogo M (2000). HIV/AIDS and ethical issues in Tanzania. East Afr Law Rev.

[12] Kohi TW, Makoae L, Chirwa M, Holzemer WL, Phetlhu DR, Uys L, Naidoo J, Dlamini PS, Greeff M (2006). HIV and AIDS stigma violates human rights in five African countries. Nurs Ethics.

[13] Herek GM, Capitanio JP (1993). Public reactions to AIDS in the United States: a second decade of stigma. Am J Public Health.

[14] Bogart LM, Wagner GJ, Galvan FH, Landrine H, Klein DJ, Sticklor LA (2011). Perceived discrimination and mental health symptoms among Black men with HIV. Cultur Divers Ethnic Minor Psychol.

[15] Earnshaw VA, Bogart LM, Dovidio JF, Williams DR (2013). Stigma and racial/ethnic HIV disparities: moving toward resilience. Am Psychol.

[16] Elford J, Ibrahim F, Bukutu C, Anderson J (2008). HIV-related discrimination reported by people living with HIV in London, UK. AIDS Behav.

[17] Schuster MA, Collins R, Cunningham WE, Morton SC, Zierler S, Wong M, Tu W, Kanouse DE (2005). Perceived discrimination in clinical care in a nationally representative sample of HIV-infected adults receiving health care. J Gen Intern Med.

[18] Galvan FH, Davis EM, Banks D, Bing EG (2008). HIV stigma and social support among African Americans. AIDS Patient Care STDS.

[19] Smith R, Rossetto K, Peterson BL (2008). A meta-analysis of disclosure of one's HIV-positive status, stigma and social support. AIDS Care.

[20] Cohen S, McKay G, Baum A, Taylor S, Singer J (1984). Handbook of psychology and health.

[21] Takada S, Weiser SD, Kumbakumba E, Muzoora C, Martin JN, Hunt PW, Haberer JE, Kawuma A, Bangsberg DR, Tsai AC (2014). The dynamic relationship between social support and HIV-related stigma in rural Uganda. Ann Behav Med.

[22] Tsai AC, Bangsberg DR, Frongillo EA, Hunt PW, Muzoora C, Martin JN, Weiser SD (2012). Food insecurity, depression and the modifying role of social support among people living with HIV/AIDS in rural Uganda. Soc Sci Med.

[23] Katz IT, Ryu AE, Onuegbu AG, Psaros C, Weiser SD, Bangsberg DR, Tsai AC (2013). Impact of HIV-related stigma on treatment adherence: systematic review and meta-synthesis. J Int AIDS Soc.

[24] Bekele T, Rourke SB, Tucker R, Greene S, Sobota M, Koornstra J, Monette L, Rueda S, Bacon J, Watson J (2013). Direct and indirect effects of perceived social support on health-related quality of life in persons living with HIV/AIDS. AIDS Care.

[25] Leserman J, Petitto JM, Golden RN, Gaynes BN, Gu H, Perkins DO, Silva SG, Folds JD, Evans DL (2000). Impact of stressful life events, depression, social support, coping, and cortisol on progression to AIDS. Am J Psychiatry.

[26] Siegel K, Lekas H-M, Schrimshaw EW (2005). Serostatus disclosure to sexual partners by HIV-infected women before and after the advent of HAART. Women Health.

[27] Kang E, Rapkin BD, Remien RH, Mellins CA, Oh A (2005). Multiple dimensions of HIV stigma and psychological distress among Asians and Pacific Islanders living with HIV illness. AIDS Behav.

[28] Heckman TG, Anderson ES, Sikkema KJ, Kochman A, Kalichman SC, Anderson T (2004). Emotional distress in nonmetropolitan persons living with HIV disease enrolled in a telephone-delivered, coping improvement group intervention. Health Psychol.

[29] Black BP, Miles MS (2002). Calculating the risks and benefits of disclosure in African American women who have HIV. J Obstet Gynecol Neonatal Nurs.

[30] Lee RS, Kochman A, Sikkema KJ (2002). Internalized stigma among people living with HIV-AIDS. AIDS Behav.

[31] Gonzalez A, Solomon SE, Zvolensky MJ, Miller CT (2009). The interaction of mindful-based attention and awareness and disengagement coping with HIV/AIDS-related stigma in regard to concurrent anxiety and depressive symptoms among adults with HIV/AIDS. J Health Psychol.

[32] Bogart LM, Wagner GJ, Galvan FH, Klein DJ (2010). Longitudinal relationships between antiretroviral treatment adherence and discrimination due to HIV-serostatus, race, and sexual orientation among African-American men with HIV. Ann Behav Med.

[33] Capron DW, Gonzalez A, Parent J, Zvolensky MJ, Schmidt NB (2012). Suicidality and anxiety sensitivity in adults with HIV. AIDS Patient Care STDS.

[34] Carrico AW (2010). Elevated suicide rate among HIV-positive persons despite benefits of antiretroviral therapy: implications for a stress and coping model of suicide. Am Psychiatric Assoc.

[35] Horberg MA, Silverberg MJ, Hurley LB, Towner WJ, Klein DB, Bersoff-Matcha S, Weinberg WG, Antoniskis D, Mogyoros M, Dodge WT (2008). Effects of depression and selective serotonin reuptake inhibitor use on adherence to highly active antiretroviral therapy and on clinical outcomes in HIV-infected patients. J Acquir Immune Defic Syndr.

[36] Chander G, Himelhoch S, Moore RD (2006). Substance abuse and psychiatric disorders in HIV-positive patients: epidemiology and impact on antiretroviral therapy. Drugs.

[37] Kalichman SC, Heckman T, Kochman A, Sikkema K, Bergholte J (2000). Depression and thoughts of suicide among middle-aged and older persons living with HIV-AIDS. Psychiatr Serv.

[38] Wonde M, Mulat H, Birhanu A, Biru A, Kassew T, Shumet S (2019). The magnitude of suicidal ideation, attempts and associated factors of HIV positive youth attending ART follow ups at St. Paul's hospital Millennium Medical College and St. Peter's specialized hospital, Addis Ababa, Ethiopia, 2018. PloS ONE.

[39] Wardell JD, Shuper PA, Rourke SB, Hendershot CS (2018). Stigma, coping, and alcohol use severity among people living with HIV: a prospective analysis of bidirectional and mediated associations. Ann Behav Med.

[40] Lancaster KE, Hetrick A, Sripaipan T, Ha TV, Hutton HE, Chander G, Latkin CA, Dowdy D, Frangakis C, Quynh BX (2020). Alcohol abstinence stigma and alcohol use among HIV patients in Thai Nguyen, Vietnam. PloS ONE.

[41] Scott-Sheldon LA, Walstrom P, Carey KB, Johnson BT, Carey MP (2013). Alcohol use and sexual risk behaviors among individuals infected with HIV: a systematic review and meta-analysis 2012 to early 2013. Curr HIV/AIDS Rep.

[42] Hendershot CS, Stoner SA, Pantalone DW, Simoni JM (2009). Alcohol use and antiretroviral adherence: review and meta-analysis. J Acquir Immune Defic Syndr.

[43] Fairbairn NS, Walley AY, Cheng DM, Quinn E, Bridden C, Chaisson C, Blokhina E, Lioznov D, Krupitsky E, Raj A (2016). Mortality in HIV-infected alcohol and drug users in St. Petersburg, Russia. PloS ONE.

[44] Samet JH, Cheng DM, Libman H, Nunes DP, Alperen JK, Saitz R (2007). Alcohol consumption and HIV disease progression. JAIDS J Acquir Immune Defic Syndr.

[45] Mekuriaw B, Belayneh Z, Molla A, Mehare T (2021). Alcohol use and its determinants among adults living with HIV/AIDS in Ethiopia: a systematic review and meta-analysis. Harm Reduct J.

[46] Sullivan LE, Saitz R, Cheng DM, Libman H, Nunes D, Samet JH (2008). The impact of alcohol use on depressive symptoms in human immunodeficiency virus-infected patients. Addiction.

[47] Mannes ZL, Dunne EM, Ferguson EG, Cook RL, Ennis N (2021). Symptoms of generalized anxiety disorder as a risk factor for substance use among adults living with HIV. AIDS Care.

[48] Brown LA, Majeed I, Mu W, McCann J, Durborow S, Chen S, Blank MB (2021). Suicide risk among persons living with HIV. AIDS Care.

[49] Stang A (2010). Critical evaluation of the Newcastle-Ottawa scale for the assessment of the quality of nonrandomized studies in meta-analyses. Eur J Epidemiol.

[50] Higgins JP, Green S (2011). Cochrane handbook for systematic reviews of interventions.

[51] Peterson J, Welch V, Losos M, Tugwell P (2011). The Newcastle-Ottawa scale (NOS) for assessing the quality of nonrandomised studies in meta-analyses.

[52] Ghiasvand H, Waye KM, Noroozi M, Harouni GG, Armoon B (2019). Clinical determinants associated with quality of life for people who live with HIV/AIDS: a Meta-analysis. BMC Health Serv Res.

[53] Ghiasvand H, Higgs P, Noroozi M (2020). Social and demographical determinants of quality of life in people who live with HIV/AIDS infection: evidence from a meta-analysis. Biodemography Soc Biol.

[54] Berger BE, Ferrans CE, Lashley FR (2001). Measuring stigma in people with HIV: psychometric assessment of the HIV stigma scale. Res Nurs Health.

[55] Sowell RL, Lowenstein A, Moneyham L, Demi A, Mizuno Y, Seals BF (1997). Resources, stigma, and patterns of disclosure in rural women with HIV infection. Public Health Nurs.

[56] Kalichman SC, Simbayi LC, Cloete A, Mthembu PP, Mkhonta RN, Ginindza T (2009). Measuring AIDS stigmas in people living with HIV/AIDS: the internalized AIDS-related stigma scale. AIDS Care.

[57] Derogatis L, Spencer P (1993). Brief symptom inventory: BSI.

[58] Marteau TM, Bekker H (1992). The development of a six-item short-form of the state scale of the Spielberger State—trait anxiety inventory (STAI). Br J Clin Psychol.

[59] Kendall CE, Boucher LM, Donelle J, Martin A, Marshall Z, Boyd R, Oickle P, Diliso N, Pineau D, Renaud B (2020). Engagement in primary health care among marginalized people who use drugs in Ottawa, Canada. BMC Health Serv Res.

[60] Brock DM, Sarason IG, Sarason BR, Pierce GR (1996). Simultaneous assessment of perceived global and relationship-specific support. J Soc Pers Relat.

[61] Sherbourne CD, Stewart AL (1991). The MOS social support survey. Soc Sci Med.

[62] Saunders JB, Aasland OG, Babor TF, de la Fuente JR, Grant M (1993). Development of the alcohol use disorders identification test (AUDIT): WHO collaborative project on early detection of persons with harmful alcohol consumption-II. Addiction.

[63] Akena D, Musisi S, Joska J, Stein DJ (2012). The association between aids related stigma and major depressive disorder among HIV-positive individuals in Uganda. PLoS ONE.

[64] Burke SE, Calabrese SK, Dovidio JF, Levina OS, Uusküla A, Niccolai LM, Abel-Ollo K, Heimer R (2015). A tale of two cities: stigma and health outcomes among people with HIV who inject drugs in St. Petersburg, Russia and Kohtla-Järve, Estonia. Soc Sci Med.

[65] Chapman Lambert C, Westfall A, Modi R, Amico RK (2020). HIV-related stigma, depression, and social support are associated with health-related quality of life among patients newly entering HIV care. AIDS Care.

[66] Courtenay-Quirk C, Wolitski RJ, Parsons JT, Gómez CA (2006). Is HIV/AIDS stigma dividing the gay community? Perceptions of HIV-positive men who have sex with men. AIDS Educ Prev.

[67] Emlet CA, Brennan DJ, Brennenstuhl S, Rueda S, Hart TA, Rourke SB (2015). The impact of HIV-related stigma on older and younger adults living with HIV disease: does age matter?. AIDS Care.

[68] Fekete EM, Williams SL, Skinta MD (2018). Internalised HIV-stigma, loneliness, depressive symptoms and sleep quality in people living with HIV. Psychol Health.

[69] Felker-Kantor EA, Wallace ME, Madkour AS, Duncan DT, Andrinopoulos K, Theall K (2019). HIV stigma, mental health, and alcohol use disorders among people living with HIV/AIDS in New Orleans. J Urban Health.

[70] Ferlatte O, Salway T (2017). Stigma and suicide among gay and bisexual men living with HIV. AIDS Care.

[71] Levi-Minzi MA, Surratt HL (2014). HIV stigma among substance abusing people living with HIV/AIDS: implications for HIV treatment. AIDS Patient Care STDS.

[72] Li Z, Hsieh E, Morano JP, Sheng Y (2016). Exploring HIV-related stigma among HIV-infected men who have sex with men in Beijing, China: a correlation study. AIDS Care.

[73] Saeidi L, Ghaedi H, Sadatamini M, Vahabpour R, Rahimipour A, Shanaki M, Mansoori Z, Kazerouni F (2018). Long non-coding RNA LY86-AS1 and HCG27_201 expression in type 2 diabetes mellitus. Mol Biol Rep.

[74] Pearson CR, Micek MA, Pfeiffer J, Montoya P, Matediane E, Jonasse T, Cunguara A, Rao D, Gloyd SS (2009). One year after ART initiation: psychosocial factors associated with stigma among HIV-positive Mozambicans. AIDS Behav.

[75] Peltzer K, Ramlagan S (2011). Perceived stigma among patients receiving antiretroviral therapy: a prospective study in KwaZulu-Natal, South Africa. AIDS Care.

[76] Rael CT, Hampanda K (2016). Understanding internalized HIV/AIDS-related stigmas in the Dominican Republic: a short report. AIDS Care.

[77] Turan B, Budhwani H, Fazeli PL, Browning WR, Raper JL, Mugavero MJ, Turan JM (2017). How does stigma affect people living with HIV? The mediating roles of internalized and anticipated HIV stigma in the effects of perceived community stigma on health and psychosocial outcomes. AIDS Behav.

[78] Turan B, Rogers AJ, Rice WS, Atkins GC, Cohen MH, Wilson TE, Adimora AA, Merenstein D, Adedimeji A, Wentz EL (2017). Association between perceived discrimination in healthcare settings and HIV medication adherence: mediating psychosocial mechanisms. AIDS Behav.

[79] Zeng C, Li L, Hong YA, Zhang H, Babbitt AW, Liu C, Li L, Qiao J, Guo Y (2018). A structural equation model of perceived and internalized stigma, depression, and suicidal status among people living with HIV/AIDS. BMC Public Health.

[80] Zhang C, Li X, Liu Y, Qiao S, Zhang L, Zhou Y, Tang Z, Shen Z, Chen Y (2016). Stigma against people living with HIV/AIDS in China: does the route of infection matter?. PLoS ONE.

[81] Zhang C, Li X, Liu Y, Qiao S, Zhou Y, Shen Z, Chen Y (2016). Substance use and psychosocial status among people living with HIV/AIDS who encountered hiv stigma in china: stratified analyses by socio-economic status. PLoS ONE.

[82] Zhang C, Li X, Liu Y, Zhou Y, Shen Z, Chen Y (2018). Impacts of HIV stigma on psychosocial well-being and substance use behaviors among people living with HIV/AIDS in China: across the life span. AIDS Educ Prev.

[83] Kleiman EM, Liu RT (2013). Social support as a protective factor in suicide: findings from two nationally representative samples. J Affect Disord.

[84] Scardera S, Perret LC, Ouellet-Morin I, Gariépy G, Juster R-P, Boivin M, Turecki G, Tremblay RE, Côté S, Geoffroy M-C (2020). Association of social support during adolescence with depression, anxiety, and suicidal ideation in young adults. JAMA Netw Open.

[85] Kim BJ, Kihl T (2021). Suicidal ideation associated with depression and social support: a survey-based analysis of older adults in South Korea. BMC Psychiatry.

[86] Turan B, Fazeli PL, Raper JL, Mugavero MJ, Johnson MO (2016). Social support and moment-to-moment changes in treatment self-efficacy in men living with HIV: psychosocial moderators and clinical outcomes. Health Psychol.

[87] Tsai AC, Bangsberg DR, Kegeles SM, Katz IT, Haberer JE, Muzoora C, Kumbakumba E, Hunt PW, Martin JN, Weiser SD (2013). Internalized stigma, social distance, and disclosure of HIV seropositivity in rural Uganda. Ann Behav Med.

[88] Maman S, van Rooyen H, Groves AK (2014). HIV status disclosure to families for social support in South Africa (NIMH project accept/HPTN 043). AIDS Care.

[89] Earnshaw VA, Chaudoir SR (2009). From conceptualizing to measuring HIV stigma: a review of HIV stigma mechanism measures. AIDS Behav.

[90] Kalichman SC, Simbayi LC, Kaufman M, Cain D, Jooste S (2007). Alcohol use and sexual risks for HIV/AIDS in sub-Saharan Africa: systematic review of empirical findings. Prev Sci.

[91] Shuper PA, Neuman M, Kanteres F, Baliunas D, Joharchi N, Rehm J (2010). Causal considerations on alcohol and HIV/AIDS—a systematic review. Alcohol Alcohol.

[92] Pence BW, Thielman NM, Whetten K, Ostermann J, Kumar V, Mugavero MJ (2008). Coping strategies and patterns of alcohol and drug use among HIV-infected patients in the United States Southeast. AIDS Patient Care STDS.

[93] Lunze K, Lioznov D, Cheng DM, Nikitin RV, Coleman SM, Bridden C, Blokhina E, Krupitsky E, Samet JH (2017). HIV stigma and unhealthy alcohol use among people living with HIV in Russia. AIDS Behav.

[94] Algarin AB, Sheehan DM, Varas-Diaz N, Fennie K, Zhou Z, Spencer EC, Cook CL, Cook RL, Ibanez GE (2021). Enacted HIV-related stigma’s association with anxiety & depression among people living with HIV (PLWH) in Florida. AIDS Behav.

[95] Demirel O, Mayda P, Yıldız N, Sağlam H, Koçak BT, Habip Z, Kadak MT, Balcıoğlu İ, Kocazeybek B (2018). Self-stigma, depression, and anxiety levels of people living with HIV in Turkey. Eur J Psychiatry.

[96] Varni SE, Miller CT, McCuin T, Solomon SE (2012). Disengagement and engagement coping with HIV/AIDS stigma and psychological well-being of people with HIV/AIDS. J Soc Clin Psychol.

[97] Algarin AB, Sheehan DM, Varas-Diaz N, Fennie K, Zhou Z, Spencer EC, Cook CL, Cook RL, Ibanez GE (2021). Enacted HIV-related stigma's association with anxiety & depression among people living with HIV (PLWH) in Florida. AIDS Behav.

[98] Parcesepe A, Tymejczyk O, Remien R, Gadisa T, Kulkarni SG, Hoffman S, Melaku Z, Elul B, Nash D (2018). HIV-related stigma, social support, and psychological distress among individuals initiating ART in Ethiopia. AIDS Behav.

[99] Wang W, Xiao C, Yao X, Yang Y, Yan H, Li S (2018). Psychosocial health and suicidal ideation among people living with HIV/AIDS: a cross-sectional study in Nanjing, China. PLoS ONE.

[100] Amiya RM, Poudel KC, Poudel-Tandukar K, Pandey BD, Jimba M (2014). Perceived family support, depression, and suicidal ideation among people living with HIV/AIDS: a cross-sectional study in the Kathmandu Valley, Nepal. PLoS ONE.

[101] McDowell TL, Serovich JM (2007). The effect of perceived and actual social support on the mental health of HIV-positive persons. AIDS Care.

[102] Mallinson RK, Rajabiun S, Coleman S (2007). The provider role in client engagement in HIV care. AIDS Patient Care STDS.

[103] Klein SJ, Karchner WD, O'Connell DA (2002). Interventions to prevent HIV-related stigma and discrimination: findings and recommendations for public health practice. J Public Health Manag Pract.

[104] Prost A, Elford J, Imrie J, Petticrew M, Hart GJ (2008). Social, behavioural, and intervention research among people of Sub-Saharan African origin living with HIV in the UK and Europe: Literature review and recommendations for intervention. AIDS Behav.

[105] Feyissa GT, Abebe L, Girma E, Woldie M (2012). Stigma and discrimination against people living with HIV by healthcare providers, Southwest Ethiopia. BMC Public Health.

[106] Li Z, Morano JP, Khoshnood K, Hsieh E, Sheng Y (2018). HIV-related stigma among people living with HIV/AIDS in rural Central China. BMC Health Serv Res.

